# Exploring antecedents of student teachers' emotions during instructional experiences: A situation‐specific analysis

**DOI:** 10.1111/bjep.12738

**Published:** 2025-02-13

**Authors:** Eva Susann Becker, Tina Hascher, Thomas Goetz, Fritz C. Staub

**Affiliations:** ^1^ Center for Teacher Education University of Cologne Cologne Germany; ^2^ Institute of Educational Science, Department of Research in School and Instruction University of Bern Bern Switzerland; ^3^ Department of Developmental and Educational Psychology, Faculty of Psychology University of Vienna Vienna Austria; ^4^ Institute of Education University of Zurich Zurich Switzerland

**Keywords:** appraisal theory, coaching, emotions, mentoring, teaching practicum, teaching quality, teacher

## Abstract

**Theoretical Background:**

In the area of teacher motivation, the teaching practicum stands out as a pivotal element. The pronounced complexity of teaching during this specific phase may pose an emotional challenge, making the exploration of student teachers' emotions a worthwhile endeavour.

**Aims:**

Based on a theoretical model and rooted in a process‐oriented perspective, this diary study examines student teachers' discrete emotions, focusing on proximal (cognitive appraisals) and distal antecedents (classroom conditions) during the teaching practicum while accounting for contextual variables.

**Sample:**

Data were collected from 178 student teachers in Switzerland and Germany and their 3736 school students. Student teachers conducted a six‐lesson‐teaching‐unit within three‐weeks of their obligatory teaching practicum (57% had prior teaching experience) and received different levels of support (coaching by peers or cooperating teachers, subject‐didactic materials, usual support).

**Methods:**

After three lessons (*N* = 511), student teachers reported their enjoyment, anger, anxiety and cognitive appraisals (control, value). School students reported on individual perceptions of class discipline and situational interest.

**Results:**

Enjoyment was strongly experienced in 80%, anger in 8% and anxiety in 14% of lessons. School students' situational interest and discipline were weakly related to enjoyment and anger, but not to anxiety. Control appraisals were strongly associated with all emotions. The frequent experience of anxiety and its lack of relation to classroom conditions deviate from findings observed in in‐service teachers.

**Relevance:**

Besides strong positive emotional experiences during the teaching practicum, the observed patterns highlight the necessity for targeted support in navigating emotional complexities during the teaching practicum.

## INTRODUCTION

Student teachers often describe the teaching practicum as the most important element in their teacher education programme and evaluate it positively across different countries and teacher education systems (Hascher et al., 2004; Kakazu & Kobayashi, 2023; Smith & Lev‐Ari, 2005). However, research indicates that university tutors or mentors rate its effectiveness lower (Perla & Agrati, 2023), and actual learning effects vary significantly due to numerous factors, such as the level of support received and its alignment with university coursework (Mok & Staub, 2021; Zeichner, 2010). Consequently, the high expectations of student teachers regarding the practicum (de Zordo et al., 2019) are not always met, corresponding to negative emotions, stress, or resignation (Hobson et al., 2008; Mahmoudi & ÿzkan, 2016; Sjølie & Østern, 2021). To reduce drop‐outs during teacher education, it is important to examine motivational‐affective processes during the teaching practicum in order to better design a learning environment that increases self‐efficacy and career choice security (Seifert & Schaper, 2018).

Taking responsibility for a class and gaining initial experiences as a teacher can be both exciting and frightening. Even a few (negative) peak experiences can influence the overall retrospective assessment of an experience (Alaybek et al., 2022; Fredrickson & Kahneman, 1993) and impact decision‐making (Angie et al., 2011). Research has shown that in an unsupervised teaching practicum, student teachers feel overwhelmed (Ronfeldt & Reininger, 2012) and numerous student teachers undertaking an initial teacher preparation programme express a range of negative emotions (Hobson et al., 2008). Recent evaluations from Germany indicate that a significant proportion of student teachers does not feel strengthened after the practicum, with about 15% even questioning their career aspirations (Becker et al., 2023).

This article extends previous findings by employing a situational assessment approach and incorporating reports from student teachers and aggregated reports from their classes to evaluate more objective situational demands. While previous studies have primarily focused on self‐reported retrospective assessments of emotions, there is a gap in understanding how specific situational factors (classroom conditions) influence these emotional experiences. By integrating both interpersonal and classroom‐level data, this study aims to provide a more comprehensive understanding of the emotional dynamics in the teaching practicum. This understanding can help teacher education programmes design interventions and support structures to better prepare and support student teachers during their practicum.

### Teacher emotions

Over the last 25 years, researchers have increasingly recognized the emotional dimension of teaching. There is accumulating scientific evidence that the emotional dimension is worth considering when talking about teaching effectiveness (Burić & Frenzel, 2023; Frenzel, 2014), teacher well‐being (Day & Qing, 2009; Hascher & Waber, 2021; Nwoko et al., 2023), job satisfaction (Ben‐Ari et al., 2003; Dreer, 2024) and turnover intentions (Lee, 2019; Li & Yao, 2022).

According to the component model of emotion (Scherer & Moors, 2019), emotions are multicomponential and have—in contrast to moods—an ‘event focus’ (Shuman & Scherer, 2014), meaning that they have a cause and are triggered by a stimulus. This stimulus is evaluated based on its significance and controllability (cognitive appraisals), which then triggers responses at various levels, including physiological reactions, behavioural tendencies and motor expressions, all contributing to the subjective feeling of the emotion.

Adapting this general emotion framework and focusing on cognitive appraisals, Frenzel and colleagues (Frenzel et al., 2009) developed a theoretical model specifically for teacher emotions in classroom interactions (see also Frenzel, 2014; Frenzel & Stephens, 2013; Keller et al., 2014). A key assumption of the model is that the cognitive appraisals of events that occur in the classroom significantly contribute to teachers' emotions. Teacher emotions, in turn, impact their teaching behaviours and student outcomes. In a school context, emotions are potentially elicited by various situations beyond classroom interactions, including administrative tasks, parent‐teacher conferences, and interactions with colleagues or school leadership (Schmidt et al., 2017). Nevertheless, teaching remains the central and most time‐consuming aspect of a teacher's professional life. For student teachers, gaining practical teaching experience is a primary goal during their practicum. Consequently, this study focuses on emotional experiences within classroom interactions, adopting the part of Frenzel's model that deals with the antecedents of teacher emotions.

Frenzel's model, informed by various theoretical assumptions, suggests that teachers generally strive for high academic performance, strong student motivation, class discipline and positive relationships with students (Butler, 2012; Tschannen‐Moran & Hoy, 2001). Classroom events that pertain to these four dimensions are particularly relevant for eliciting emotions. In a qualitative study (de Ruiter et al., 2019), relational or social–emotional student behaviours—such as hostility or aggression towards teachers or peers, lack of discipline or positive interactions like friendly chats or shared jokes—were most frequently cited by teachers as relevant classroom events for both, their positive and negative emotional reactions. Student motivation (engagement or disengagement) was predominantly mentioned if it represented an atypical behaviour for a student. Achievement behaviours (e.g. a student understands the materials after first difficulties, or a student receives a good grade) was mainly linked to teachers' positive emotional reports. This is in line with quantitative findings showing that student misbehaviour is a major cause of teachers' anger and stress experiences (Chang & Davis, 2009; Tsouloupas et al., 2010) and that learning successes can evoke joy and pride in teachers (Frenzel, 2014; Pawłowska, 2020).

Our study focuses on classroom events that student teachers encounter in every lesson, emphasizing factors that are observable within the limited timeframe of a teaching practicum. Specifically, we assess two dimensions: situational interest (as an indicator of student motivation) and discipline (as an indicator of social–emotional behaviour). Both dimensions have been shown in previous studies to significantly relate to teacher emotions (Becker et al., 2015; Becker & Keller, 2022; Chang, 2013; de Ruiter et al., 2020; Frenzel et al., 2009).

To better understand student teachers' emotional experiences, this study considers both classroom conditions as reported by school students and the student teachers' experiences within the same situations. This dual approach helps to disentangle actual classroom events from teachers' cognitive appraisals. It is not the situation itself that triggers an emotional response, but the teacher's interpretation of it, that is their perceptions and judgements of situational occurrences, which are shaped by individual factors (see e.g. Roseman & Smith, 2001). The most widely recognized cognitive appraisals related to emotions in an academic context involve the level of controllability of actions and outcomes and the value of these actions and outcomes (Pekrun & Perry, 2014). In this study, lesson‐specific classroom conditions reported by the whole class serve as distal antecedents to emotions, while student teachers' control and value appraisals following the lesson are treated as proximal antecedents.

### Emotions in the teaching practicum

The teaching practicum and its conditioning factors for successful learning, as well as its relations to self‐efficacy, and student teachers' professional identity development have been investigated in numerous studies (Huang & Wang, 2024; Lawson et al., 2015; Mena et al., 2023). However, emotions have so far played a comparatively minor role in this field of research (Porsch, 2018).

Given the essential role of the teaching practicum in teacher education systems and the high value and expectations student teachers place on it, it is anticipated that student teachers experience strong emotions during this period. Although some quantitative studies have shown that predominantly positive emotions are experienced or expected during the teaching practicum (de Zordo et al., 2019; Timoštšuk et al., 2016), qualitative findings indicate that negative emotions, especially anxiety, are also present and can significantly influence student teachers' perception of the teaching practicum (Hastings, 2004; Timoštšuk & Ugaste, 2012). Students take on temporary responsibility for a class and often feel inadequately prepared (Hoferichter, 2019). Additionally, the complexity of teaching becomes particularly evident during these initial teaching experiences (Sutton & Wheatley, 2003).

This study focuses on three discrete emotions—enjoyment, anxiety and anger—that have been frequently examined in the context of teacher emotions (e.g. Frenzel et al., 2016, 2020) and also specifically within the teaching practicum (e.g. Bach & Hagenauer, 2022). Enjoyment is inherently tied to teacher motivation and can reinforce a student teacher's commitment to the profession (Li & Yao, 2022). Anxiety, on the other hand, often arises in challenging, unfamiliar and uncontrollable situations during the practicum and can diminish teaching effectiveness in the short term and undermine career‐choice security in the long term (Agyapong et al., 2022; Porsch & Gollub, 2018). Finally, anger can emerge when high expectations are unmet or when disruptions and challenges to authority arise within the classroom (Chang, 2013). Anger has been shown to impact overall practicum performance negatively (Chen, 2019). Intense negative emotional experiences characterize or bias the retrospective assessment of events (Gorin & Stone, 2001; Timoštšuk et al., 2016). In the context of the teaching practicum, such a bias may negatively influence career decisions (Daniels et al., 2006; Krannich et al., 2019; Wigfield et al., 2002), as student teachers’ emotions are related to self‐concept and self‐efficacy (Bach & Hagenauer, 2022).

### Dual role of student teachers in the teaching practicum

Beyond the anticipation of strong emotions due to high values and expectations associated with the teaching practicum, there are additional reasons why student teachers' emotional experiences may differ from those of in‐service teachers (Ji et al., 2022). Student teachers juggle dual roles as both teacher and student, being in a transitional phase between a student's and a teacher's habitus (Kahlau, 2023), a situation that introduces further emotional antecedents. In adapting Frenzel's model, it can be considered that a student teacher—in the role of a student—is also potentially influenced by the actions of mentor teachers (e.g. a school‐based cooperating teacher), similar to how school students are affected by their teacher's instructional behaviours. Mentoring in the teaching practicum serves three main functions: cognitive, relational and affective support (Wang et al., 2025). However, an array of studies shows that some mentor teachers may fall short of providing this support, whether due to being ‘unavailable’ or adopting a ‘tough’ approach (for an overview, see Thornton, 2024). This variability might stem from a lack of formal preparation, as there is often no mandatory programme for mentor teachers. Even where programmes exist, they seldom address emotional support explicitly, and the evidence on the effects of various mentor preparation methods is sparse and underdeveloped (Maxwell et al., 2024). While meta‐analytic findings suggest that cognitive modelling of lesson planning and teaching has small to moderate effects on student teachers' instructional skills (Mok & Staub, 2021), there are no accumulated findings regarding mentor teachers' role in providing emotional and psychological support nor their impact on fostering emotional and motivational aspects of teaching. In an exploratory approach, this study aims to examine if and how the support received by formally trained persons shape student teachers' emotional experiences during the practicum.

### The role of previous teaching experiences

A teacher shortage in German‐speaking countries has led to the increased hiring of non‐traditionally certified teachers and student teachers as in‐service teachers (Hascher & Krummenacher, 2024; Meyer et al., 2024; Richter et al., 2024), a trend that can be seen globally (OECD, 2021; Sutcher et al., 2019). In these positions, student teachers as in‐service‐teachers are faced with demanding tasks and responsibilities such as teaching independently, assigning grades or conducting parent‐teacher conferences (Simonis & Klomfaß, 2023). Teaching experiences gained outside formal teacher education programmes are usually not supervised by university mentors and often also not by school‐based cooperating teachers as teacher shortage and high workload has led to the job hiring in the first place (Meyer et al., 2024). Hence, stress experiences and deprofessionalization are discussed as possible outcomes (Kahlau, 2023; Simonis & Klomfaß, 2023).

However, student teachers working as in‐service teachers have also reported high levels of social support from colleagues, which buffered the negative effects of stress experiences (Meyer et al., 2024). In such cases, these practical experiences may help reduce unfamiliarity with various classroom situations during the teaching practicum, potentially lowering anxiety and enhancing feelings of control. This may bring student teachers' emotional experiences closer to those of in‐service teachers.

The present study aims to explore whether prior teaching experiences outside the formal curriculum influence student teachers' emotions when teaching occurs under the formal guidance of a cooperating teacher.

### The present study

The aim of this study is to investigate the emotions enjoyment, anger and anxiety and the antecedents of these emotions during initial teaching experiences in the teaching practicum. We include distal antecedents of classroom conditions using aggregated reports from the class (school students' reports on class discipline and situational interest) and proximal antecedents using student teachers' self‐reports on cognitive appraisals (sense of control and value). In addition, we are also exploratively investigating the role of contextual variables (previous teaching experiences and support structure) of the teaching practicum.

Our research questions are as follows:

RQ1: What emotions do student teachers experience during the lessons?

Hypothesis 1a: Based on previous empirical findings (e.g. Bach & Hagenauer, 2022; Frenzel, 2014; Ji et al., 2022), we expect that joy is the predominant emotion, followed by anxiety (or nervousness) and anger.
Hypothesis 1b: Based on intra‐individual research on academic emotions (e.g., Goetz et al., 2019; Nett & Goetz, 2011), we expect between‐lesson‐fluctuations in emotion experiences.

RQ2: How strongly are the experienced emotions related to the classroom situation and to student teachers' cognitive appraisals

Based on Frenzel's' model on teacher emotions and previous empirical studies (Becker et al., 2015), we expect,
Hypothesis 2a: a significant relation between classroom conditions and student teachers' emotions;
Hypothesis 2b: a significant relation between cognitive appraisals and emotions;
Hypothesis 2c: that the relation between classroom conditions and emotions is partially mediated by cognitive appraisals.

RQ3: Which contextual variables are related to students' cognitive appraisals and emotions?

We expect heterogeneity among student teachers regarding previous teaching experiences. We exploratively investigate how such experiences relate to emotional experiences and their antecedents. Additionally, we investigate the role of support structures during the teaching practicum by comparing groups of student teachers who received systematically different levels of support (within the framework of a larger intervention study). Since none of the support variations explicitly targeted emotions or emotional support, we do not expect substantial effects on emotions or their antecedents. However, we account for potential intervention effects by including dummy variables for the intervention conditions as covariates in our analyses (see Statistical analysis section).

## MATERIALS AND METHODS

### Sample

The sample comprised 178 student teachers (68% female, *M*_age_ = 26.92 years), recruited from four teacher education institutes in Switzerland and Germany. Each student teacher was completing an obligatory teaching practicum in the subject domain German (for 53% it was their first teaching practicum in this domain) and was assigned to a school in rural, suburban or metropolitan areas, covering secondary I and II levels. During the practicum, each student teacher taught a 6‐lesson unit on “written argumentation” in German to 7th–10th graders. Data were also collected from 3736 students (52.1% female, *M*_age_ = 14.85 years).

### Procedure

The conditions of a teaching placement vary greatly, both between and within institutions. To ensure a minimum of comparability among practicum experiences, they had these common features: (1) They were of at least three weeks in duration, (2) were supervised by a cooperating teacher and (3) included six lessons of teaching the subject German to a 7th–10th grade class (time span between first and sixth lesson: *M* = 12.21 days, *SD* = 5.95 days). After three out of the six lessons, both student teachers and their classes completed paper‐pencil questionnaires. Student teachers reported on their teaching‐related emotions and cognitive appraisals, while school students documented class discipline and situational interest during the same lessons. Out of 534 expected lessons, 511 lessons were documented by teachers, and 506 by school students, resulting in a strong response rate of 94.75%, which is notable for diary studies.

This study was conducted as part of the larger research project COPRA investigating support structures during the teaching practicum, with systematic variations in the support provided to student teachers. Data were collected in three waves from 2016 to 2019. In the first wave (2016–2017), student teachers at four teacher education institutes received the usual support without specific instructions (*n* = 75). In subsequent waves (2017–2019), student teachers were randomly assigned to one of three groups:

1. Teaching materials with usual support: Participants received non‐binding subject‐specific didactic materials developed by experts alongside general support (*n* = 33).
2. Teaching materials and peer coaching: Participants received didactic materials and peer support from another student teacher trained in Content‐Focused (Peer) Coaching (Kreis & Staub, 2017) through a 4‐h workshop (*n* = 27).
3. Teaching materials and coaching by the cooperating teacher: Participants received didactic materials and additional support from a cooperating teacher trained in Content‐Focused Coaching (CFC, *n* = 49).

Further information on CFC and a detailed description of the employed intervention can be found elsewhere (Becker & Staub, 2018; Staub et al., 2003). Group sizes varied due to institutional constraints, as peer coaching required two student teachers with similar study backgrounds to be placed at the same school. This arrangement was feasible at only two out of four institutions: one in Germany and one in Switzerland.

### Ethics statement

The study adhered to ethical standards by the Federation of German Psychologists Association (2005) and the American Psychological Association (2010). It was approved by the ethics board of the lead university in Switzerland (University of Zurich, approval no. 16.6.7) and by the ministry for science and arts in Bavaria, Germany (approval no. X.7‐BO4106.2016/28/6). Written informed consent was obtained by all study participants.

### Measures

#### Student teachers' self‐reported emotions

Student teachers' lesson‐related emotions were measured with single items that are based on the Teacher Emotion Scales (Frenzel et al., 2016) and previously conducted diary studies (Becker et al., 2015). The item prompt was adapted to suit the momentary assessment approach and were formulated as follows: “In this lesson, I enjoyed teaching”, “In this lesson I felt angry”, “In this lesson I was tense and nervous”, and they were rated on a 5‐point intensity scale from (1) not at all to (5) very strongly. Single items are a regular approach in diary studies to reduce invasiveness (Ohly et al., 2010) and have been shown to show reliable results for measures of affective states (Gogol et al., 2014).

#### Student teachers' self‐reported control and value appraisals

Student teachers' control appraisals were assessed with four items referring to student teachers' general appraisal of control and their perceived instructional competence: General appraisal of control was assessed with previously employed items (“In this lesson, I felt I had everything under control”, see Becker et al., 2015) and control over instructional practices was assessed with a modified subscale for understandability from the COACTIV‐study (“In this lesson, I have explained well”, see Baumert et al., 2009). Items were rated on a 5‐point Likert scale from (1) strongly disagree to (5) strongly agree and internal consistency was assessed with Cronbach's Alpha for each of the three assessment points (Mean *α* = .82).

Value appraisals were assessed with three items (Mean *α* = .60), one general value appraisal (“In this lesson it was very important to me to teach well”) and two modified items from the COACTIV‐study referring to intrinsic value (e.g. “In this lesson, the subject matter was of great personal importance to me.”).

#### Student‐reported classroom conditions

Situational interest was assessed using a four‐item measure based on Tsai et al. (2008) and items were rated on a 5‐point Likert scale from (1) strongly disagree to (5) strongly agree (e.g. “In this lesson I enjoyed the topic”). The scale showed high internal consistency (Mean *α* = .94). Mean scale scores were calculated and then aggregated at the lesson level, resulting in 506 ratings from *M* = 18.80 school students. This aggregation was justified by sufficient homogeneity among students as indicated by intraclass coefficients (ICC; ICC(1) = .20, ICC(2) = .82), making these scores reliable indicators of classroom conditions (Lüdtke et al., 2006).

School students discipline was assessed with a two‐item‐measure based on the COACTIV‐study referring to classroom disruptions and effective use of time (Baumert et al., 2009). Both items were reverse coded before further analyses so that high values indicate high discipline. The scale showed good internal consistency (Mean *α* = .89) and homogenous reports among the students from one class for each lesson (ICC(1) = .20, ICC(2) = .83).

#### Student teachers' prior teaching experiences

Student teachers' prior teaching experience was assessed with the single item: “Do you have any teaching experience that was gained outside of the formal teacher education programme?” If they answered yes, they were asked about the duration (in weeks) and the number of lessons taught per week. Among the participants, 96 student teachers reported prior teaching experience (57.20%), ranging from a few lessons to 13 years of experience as a full‐time teacher (range: 2–16,794 lessons, Median = 50 lessons taught outside the formal teacher education programme). Preliminary analyses showed similar correlations between the binary variable “teaching experience” (yes/no) and the amount of experience with our study variables. To simplify the model, we used only the binary indicator of teaching experience (*prev. TExp*).

#### Support structures in the teaching practicum

Dummy variables were used to control and evaluate the relations between the different levels of formalized support that student teachers received during their teaching practicum and our study variables. The baseline assessment (usual support without specific instructions, *UsualSup*) served as the reference group so that three dummies – teaching materials with usual support (*Mat. + UsualSup*), peer coaching (*Mat. + Peer*), coaching by cooperating teacher (*Mat. + Coach*) were entered as Level‐2 covariates.

### Statistical analysis

All analyses were conducted in *Mplus* version 8.2 (Muthén & Muthén, 1998–2017) using robust maximum likelihood estimation. The collected data represent a nested data structure with three repeated measurements (Level 1: *N* = 511 lessons) within persons (Level 2: *N =* 178 student teachers). ICCs were calculated using intercept‐only models in *Mplus* to determine the percentage of variance attributable to both levels of analysis (Hox et al., 2018; Woehr et al., 2015). To account for the nested structure, we analysed our data, which included both observed and latent variables, using multilevel structural equation modelling (*MSEM*). We employed concurrent models at both the within and between levels. Within‐person effects can be interpreted as being controlled for their between‐level variation (Preacher et al., 2010).

Missing data for our study variables ranged from 5.2% to 7.5%. As the missing data were not related to our study variables, we employed full information maximum likelihood (FIML) estimation, which assumes that data are missing at random (MAR) and utilizes all available data to derive parameter estimates. For all analyses, a *p*‐value of < .05 was used as the threshold for statistical significance. Model fit was assessed using the following indices: chi‐square, Root Mean Square Error of Approximation (*RMSEA*), Comparative Fit Indes (*CFI*) and Standardized Root Mean Square Residual (*SRMR*_within_). We followed conventional cut‐off criteria, that is *CFI* values ≥ 0.90 or ≥ 0.95 and *SRMR* and *RMSEA* scores lower than .08 reflect an acceptable fit (Hu & Bentler, 1999; Little, 2013).

To examine the factorial structure of our measures, we conducted multilevel confirmatory factor analysis. The results indicated that the four‐factor model, which distinguishes between class discipline, class motivation, student‐teacher control and student‐teacher value, provided a significantly better fit to the data compared to a two‐factor model (with classroom conditions and appraisals), Δ*χ* ^2^(10) = 511.53, *p* < .001. The four‐factor model demonstrated a good fit to the data: *χ* ^2^(118) = 246.34, *RMSEA* = .05, *CFI* = .96, *SRMR*_within_ = .05, thereby supporting the discriminant validity of the constructs.

## RESULTS

### Student teachers' emotions in the practicum

Descriptive statistics and ICCs are displayed in Table 1. The results confirm *Hypothesis 1a*, that is enjoyment was the most intensely experienced emotion among student teachers (*M* = 4.07), followed by anxiety (*M* = 2.25) and anger (*M* = 1.79). A frequency analysis further revealed that in 79.9% of the lessons analysed, student teachers reported intense enjoyment (≥ 4 on a five‐point scale), while intense anxiety/nervousness and anger were reported in 14.1% and 7.8% of the lessons, respectively. Supporting *Hypothesis 1b*, ICCs show that the majority of variance in all three emotion experiences was between lessons. The highest fluctuation between‐lessons was found for enjoyment (ICC_between_ = .29, i.e., 71% of variance lies between lessons) and anger (ICC_between_ = .30) followed by anxiety (ICC_between_ = .44).

**TABLE 1:** Descriptive statistics, ICCs and intercorrelation of all study variables.

Variable	1	2	3	4	5	6	7	8	9	10	11	12
1. ST anger		.47**	−.82**	−.79**	−.35**	−.29*	−.37**	−.10	.10	.05	−.04	−.14
2. ST anxiety	.14*		−.47**	−.81**	−.08	−.13	.09	.12	−.08**	−.02	−.11*	−.32**
3. ST enjoyment	−.44**	−.16**		.82**	.71**	.40**	.40**	−.04	.09	.01	−.12*	.06
4. ST control	−.40**	−.37**	.50**		.47**	.25*	.14	.00	.00	−.13	−.02	.23*
5. ST value	−.11	−.04	.28**	.25**		.04	.23*	.13*	−.03	.03	−.05	.10
6. Class motivation	−.17**	−.06	.20**	.14**	.15*		.50**	−.00	.21**	.07	−.04	−.04
7. Class discipline	−.16**	−.03	.23**	.16**	.01	.27**						
8. Mat. + Coach												
9. Mat. + Peer												
10. Mat. + UsualSup												
11. UsualSup												
12. Prev. TExp.												
*M*	1.79	2.25	4.07	3.83	3.98	3.58	3.84	0.26	0.15	0.19	0.40	0.58
Var	1.01	1.24	0.70	0.38	0.39	0.21	0.21	0.19	0.31	0.15	0.24	0.26
Min	1.00	1.00	1.00	1.75	1.50	2.09	2.15	0.00	0.00	0.00	0.00	0.00
Max	5.00	5.00	5.00	5.00	5.00	4.55	4.87	1.00	1.00	1.00	1.00	1.00
ICC	.30	.44	.29	.38	.49	.71	.63					
*N*	485	483	487	489	488	495	495	178	178	178	178	167

*Note*: *N* = 178 student teachers, number of lessons = 511; all within‐variables 1–7 were rated on 5‐point‐Likert‐Scales. Between‐variables 8–12 are dummy coded (1 = yes). Below the diagonal are the within correlations with group‐mean‐centred predictors to depict intra‐individual correlations. Above the diagonal are the between correlations at the person level.

* *p* < .05.

** *p* < .01.

### Antecedents of student teachers' emotions

To examine *Hypothesis 2a*, that is the relationship between classroom conditions (reported by school students) and student teachers' emotions, we conducted three *MSEM*, one for each emotion (enjoyment, anxiety and anger). Classroom conditions were modelled as two latent variables—class motivation and class discipline—at both the within‐ and between‐person levels. While our primary interest was in within‐person relationships, modelling at both levels allowed us to account for between‐person variations. The standardized coefficients and explained variance at the within‐person level are presented in Table 2.

**TABLE 2:** Student teachers' emotions predicted by classroom conditions (within‐level).

Classroom conditions	Enjoyment		Anger		Anxiety	
	*β*	SE	*β*	SE	*β*	SE
Class motivation	.14*	.06	−.10	.07	−.04	.07
Class discipline	.20**	.07	−.15*	.07	−.01	.07
*R* ^2^	.08**		.04		.00	

*Note*: Independent variables were modelled as latent variables. Classroom conditions were correlated with each other. All relations were modelled on the within and between level. *R* ^2^ refers to the explained variance on the within level. Model fit for the respective models was as follows: Enjoyment: *χ* ^2^ = 66.22, df = 24, *p* = .00, *RMSEA* = .06, *CFI* = .98; *SRMR*_within_ = .03; Anger: *χ* ^2^ = 70.41, df = 24, *p* = .00, *RMSEA* = .06, *CFI* = .98, *SRMR*_within_ = .03; Anxiety: *χ* ^2^ = 53.01, df = 24, *p* = .00, *RMSEA* = .05, CFI = .99, *SRMR*_within_ = .02.

* *p* < .05.

** *p* < .01.

Overall, the relationships between classroom conditions and student teachers' emotions were weak. Notably, there was no significant relationship between classroom conditions and anxiety. The strongest relationship was observed for enjoyment, where classroom conditions explained 8% of the variance at the within‐person level. However, for anxiety and anger, the explained variance at the within‐person level was not significant. Hence, *Hypothesis 2a* is only partially confirmed.

**FIGURE 1:**
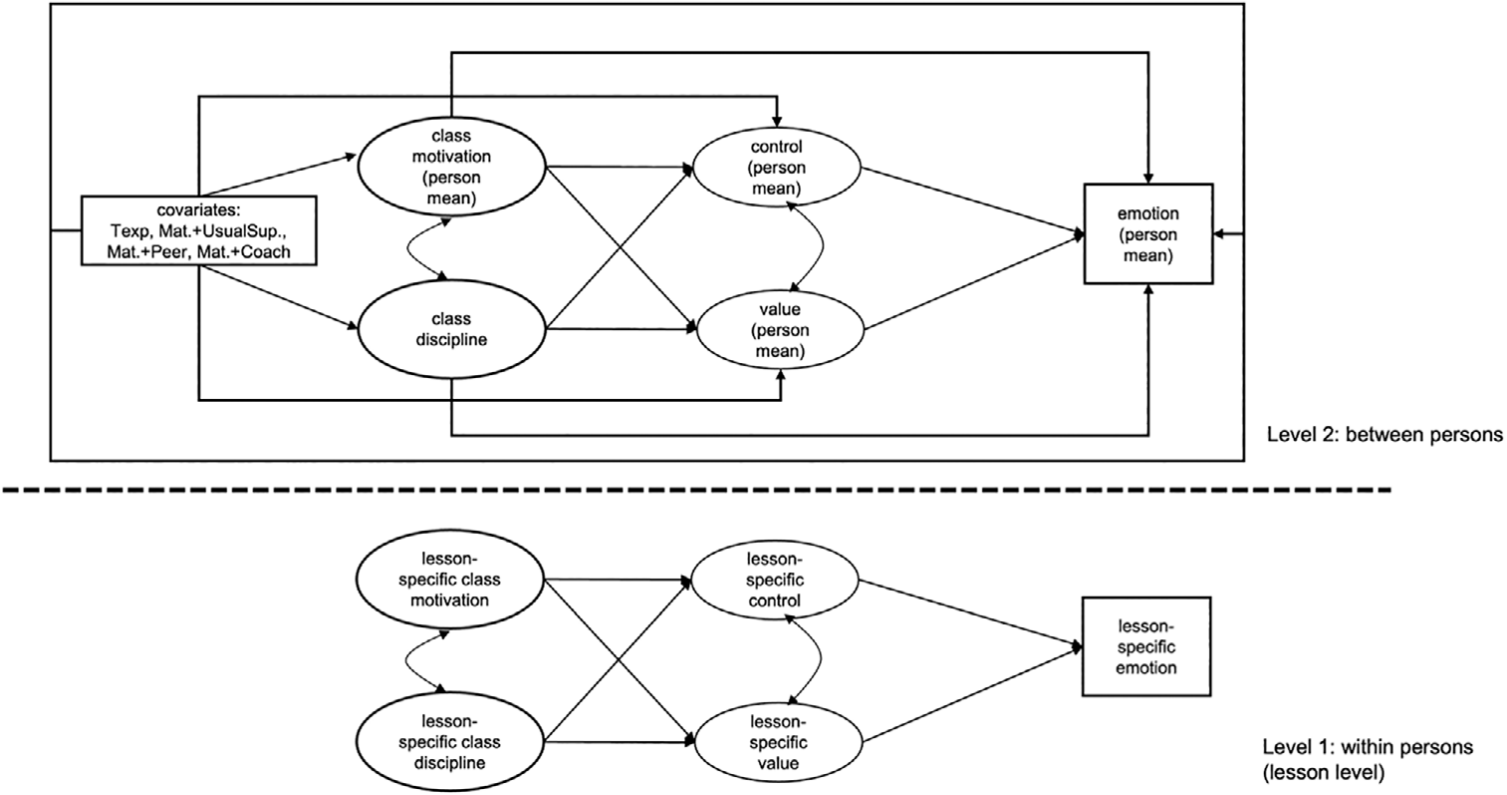
Figural representation of the multilevel structural equation models (full model) with both level of analysis and covariates depicted.

**FIGURE 2:**
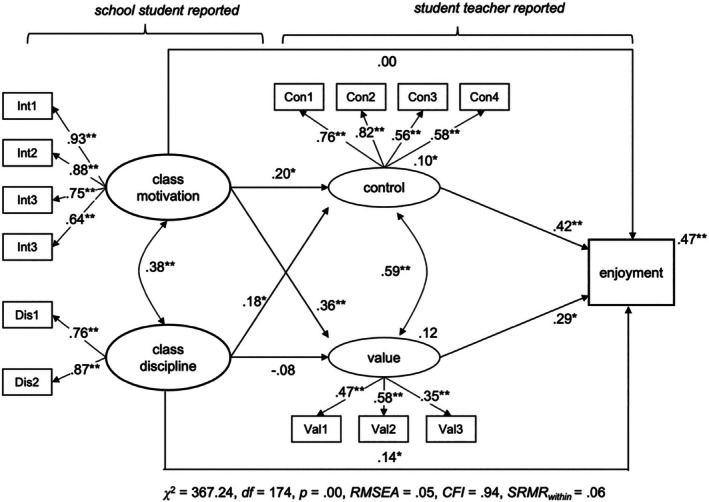
Classroom conditions and cognitive appraisals predicting teacher enjoyment with partial mediation. MSEM—within persons; regression coefficients for the latent variables are shown and standard coefficients between the latent and manifest variables. Estimates at the dependent variable represent explained within‐level variance (*R* ^2^). *p < .05. **p < .01. *p < .05. **p < .01. Total/total indirect effect from class motivation to enjoyment (*β*_direct_ = .19, *p* = .00; *β*_indirect_ = .19, *p* = .01), total/total indirect effect from class discipline to enjoyment (*β*_direct_ = .20, *p* = .01; *β*_indirect_ = .05, *p* = .46). *p*‐values are reported because bootstrap‐CIs are not available in two‐level‐models in *Mplus*.

**FIGURE 3:**
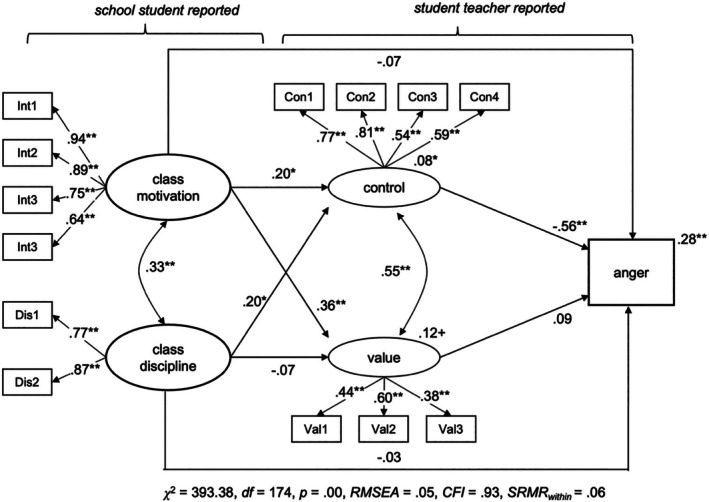
Classroom conditions and cognitive appraisals predicting student teacher anger. MSEM—within‐persons; regression coefficients for the latent variables are shown and standard coefficients between the latent and manifest variables. Estimates at the dependent variable represent explained within‐level variance (*R* ^2^). *p < .05. **p < .01. Total/total indirect effect from class motivation to anger (*β*_direct_ = −.15, *p* = .07; *β*_indirect_ = −.08, *p* = .18), Total/total indirect effect from class discipline to anger (*β*_direct_ = −.14, *p* = .03; *β*_indirect_ = −.11, *p* = .09). *p*‐values are reported because bootstrap‐CIs are not available in two‐level‐models in Mplus.

**FIGURE 4:**
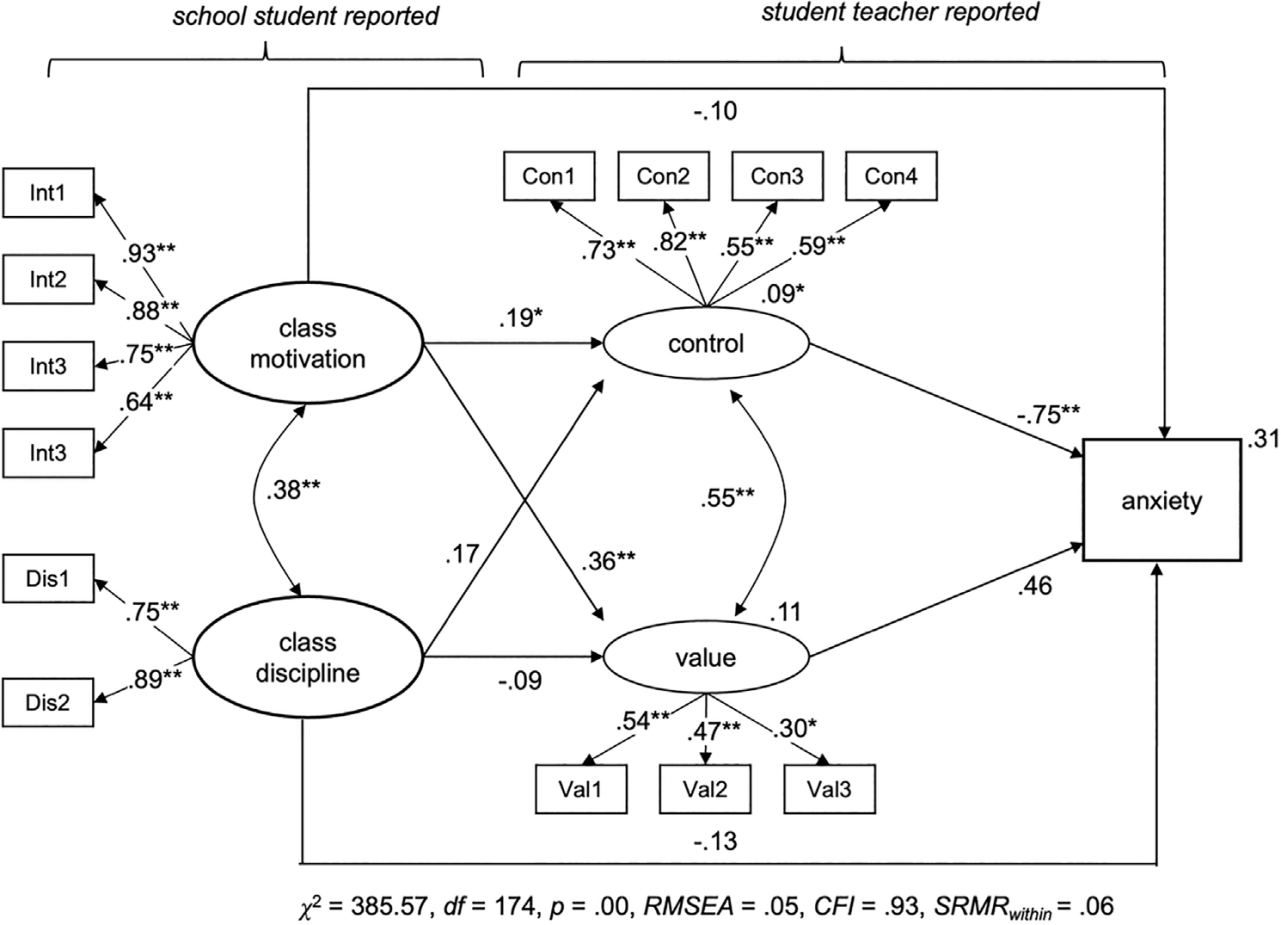
Classroom conditions and cognitive appraisals predicting student teacher anxiety. MSEM—within‐persons; regression coefficients for the latent variables are shown and standard coefficients between the latent and manifest variables. Estimates at the dependent variable represent explained within‐level variance (*R* ^2^). *p < .05. **p < .01. Total/total indirect effect from class motivation to anxiety (*β*_direct_ = −.08, *p* = .24; *β*_indirect_ = .02, *p* = .85), Total/total indirect effect from class discipline to anxiety (*β*_direct_ = −.04, *p* = .55; *β*_indirect_ = −.17, *p* = .11). *p*‐values are reported because bootstrap‐CIs are not available in two‐level‐models in Mplus.

To assess *Hypothesis 2b* and *Hypothesis 2c*, that is the role of cognitive appraisals, we conducted three additional *MSEM* models (full model), again one for each emotion. Classroom conditions and cognitive appraisals were modelled as two latent variables each, with the latent factors allowed to correlate. The model was specified so that classroom conditions predicted student teachers' appraisals, which in turn predicted their emotions. As we assumed partial mediation, we tested for direct and indirect effects of classroom conditions on student teachers' emotions at the within‐level. To control for between‐level variation of our study variables, we employed a concurrent model at Level 2 that also included our covariates (context variables, i.e., prior teaching experiences and level of support). The structural model of the MSEM is depicted in Figure 1, while the results are shown in Figures 2, 3, 4.

Student teachers' anger, anxiety and enjoyment were significantly predicted by control appraisals, partially supporting *Hypothesis 2b*. Teachers who reported a higher sense of control over their instructional practice within a lesson also reported more enjoyment (*β* = .25, *p* < .01) and less anger (*β* = −.56, *p* < .01) and anxiety (*β* = .75, *p* < .01). Conversely, value appraisals, which reflect the importance student teachers place on the lesson content and their desire to deliver a good lesson, were not significantly related to any of the emotions. Explained variance at the within‐level increased from 0% to 31% for anxiety, 4% to 28% for anger and 8% to 47% for enjoyment when appraisals are included.

Results from direct and indirect effects indicate that appraisals partially mediated the relationship between classroom conditions and emotions, which is in line with *Hypothesis 2c*. However, these findings should be interpreted with caution due to the weak relationship between classroom conditions and student teachers' negative emotions.

### Relations to student teachers’ previous teaching experiences and support structures during the teaching practicum

To evaluate RQ3, we first considered between‐person correlations (Table 1) that indicated that student teachers who received *usual support* at their school experienced less intense anxiety (*β* = −.11, *p* < .05) but also reported lower levels of enjoyment (*β* = −.12, *p* < .05) compared to the three other groups. Student teachers who were coached by a trained cooperating teacher reported higher value appraisals during teaching (*β* = .13, *p* < .05). Those coached by a trained peer reported lower levels of anxiety (*β* = −.08, *p* < .01) and their classes demonstrated higher motivation (*β* = .21, *p* < .01). Furthermore, previous teaching experiences correlated with lower levels of anxiety (*β* = −.29, *p* < .01) and higher levels of control appraisals (*β* = .22, *p* < .01).

Since we had no specific hypotheses regarding the role of contextual variables, they mainly served as covariates in our full model (see Figure 1). We evaluated the standardized coefficients for the intervention groups on classroom conditions, appraisals and emotions separately. Results mainly matched those obtained from bivariate correlations. At the person‐level (between‐level), student teachers with previous teaching experiences reported slightly higher control appraisals (*β* = .20, *p = *.06) and lower anxiety (*β* = −.19, *p* < .05) throughout their teaching practicum. Students that were coached by a formally trained cooperating teacher reported higher anxiety scores (*β* = .24, *p* < .05) and school students being taught by a student teacher who participated in the peer‐coaching‐group, reported higher motivation scores (*β* = .25, *p* < .01).

## DISCUSSION

The current study offers insights into the emotional experiences of student teachers during their practicum, highlighting that the teaching practicum is not a universally positive experience. Approximately 14% of the lessons were marked by intense nervousness, a figure that suggests feelings of being overwhelmed among some student teachers. This frequency of intense nervousness is notably higher than that reported by experienced teachers (Frenzel, 2014), indicating that student teachers face emotional challenges during teaching more often.

The presence of anxiety, while somewhat concerning, might also serve as a catalyst for growth, prompting student teachers to recalibrate their expectations and identify areas for professional development. It is unrealistic to expect a completely anxiety‐free teaching practicum; instead, the goal should be to manage and learn from these emotions. Previous research has linked emotional experiences, both positive and negative, to the development of self‐efficacy among student teachers (Bach & Hagenauer, 2022). Self‐efficacy, or the belief in one's ability to succeed, has been found to relate to the commitment to the teaching profession and intention to quit among student teachers (Chesnut & Burley, 2015; Pfitzner‐Eden, 2016; Westphal et al., 2024). Therefore, it is essential that the emotions experienced during the practicum are productively addressed through reflective practices.

While a main goal of formal coaching and mentoring‐approaches is to offer emotional support (Hobson et al., 2009; Lawson et al., 2015), our study showed that this is not always the case. On the contrary, student teachers that were coached by a formally trained cooperating teacher reported even higher anxiety levels throughout their practicum. Our training for cooperating teachers and peer students is based on CFC (for more information see Becker et al., 2019; Kreis & Staub, 2017) which emphasizes joint lesson planning in a dialogic style. This approach provides student teachers with a setting where they can discuss teaching‐related questions more openly, without the strong evaluative component typically present in post‐lesson conferences. However, joint lesson planning may inadvertently increase pressure on student teachers to perform well during the actual lesson. To address this, trust‐building measures and a clear rationale for joint lesson planning should be more strongly integrated into the training. Moreover, the training as implemented in this study did not explicitly target emotional outcomes or strategies for managing emotions; a limitation shared by other prominent coaching and mentoring programmes (Bradbury, 2010; Vermunt et al., 2019). Future coaching models could benefit from incorporating components that offer emotional support and help student teachers develop effective coping strategies for managing their emotions.

An important finding in that regard is that the emotions experienced by student teachers during the practicum were only weakly related to school students' academic behaviours during lessons. Instead, emotions were more strongly predicted by student teachers' individual appraisals, particularly their sense of control. This suggests that student teachers might adopt an internal attention focus, focusing less on external classroom dynamics. This finding is in line with eye‐tracking studies focussing on student teachers' attention allocation (Chaudhuri et al., 2022; van den Bogert et al., 2014). Guided reflection sessions, whether with peers or cooperating teachers, could therefor aim to categorize emotional experiences and discuss possible regulation strategies, such as cognitive reappraisal (Denny & Ochsner, 2014; Gross, 2014). Future research should explore effective interventions in the practicum setting that strike a balance between exposing student teachers to the complexities of teaching and reducing overwhelming anxiety. If our findings replicate in other studies, it may also be necessary to adapt the theoretical model on the antecedents of teacher emotions for the group of student teachers in the teaching practicum. Emotions seem to be elicited by more or other variables as proposed in the theoretical model.

Despite the valuable insights provided by this study, several research gaps remain. For example, little is known about how the emotional challenges faced during student teaching affect long‐term teaching competence and career outcomes. Our study only accompanied student teachers over a relatively short period of time (3 weeks). It would be interesting to investigate how these emotional experiences change over time in longer‐lasting field experiences. A previous study from Germany with pre‐ and post‐measurements already showed that anxiety decreases over the course of a 6‐month teaching practicum (Porsch & Gollub, 2018). Also, our study shows that student teachers with teaching experiences gained outside of formal teacher education programmes, experience less anxiety. Longitudinal studies with repeated momentary assessments throughout the practicum could assess if the relations between situational demands and emotion experiences develop over time and capture intraindividual differences in practicum experiences (Jähne et al., 2022). Additionally, more research is needed on how student teachers can effectively manage their emotions during their practicum. Understanding these dynamics could lead to improvements in preparing student teachers for the emotional challenges of the teaching profession.

Finally, the relationship between teachers and school students is crucial in the context of teacher emotions. Our study did not assess relational variables, as the student teachers were all relatively new to their classes. Interventions that foster the relationship between student teachers and school students—such as allowing more time for cultivating emotional bonds (Romo‐Escudero et al., 2023) or focusing more explicitly on school students experiences in pre‐ and post‐lesson reflection, as it is suggested in lesson‐study approaches (Cajkler et al., 2013; Vermunt et al., 2019)—may be helpful in strengthening the connection between student teachers and their school students in the classroom.

### Strengths and limitations

This study employed a situational sampling approach, which allowed for the collection of real‐time data on student teachers' emotional experiences. This method provides a more accurate depiction of emotions as they occur in natural settings, as opposed to relying solely on retrospective self‐reports, which are prone to recall bias. Additionally, this approach offers insights into the variability of emotions over time and across different situations, enhancing our understanding of the dynamics of emotional experiences.

A further strength of this study is the inclusion of data from multiple sources. By focusing on classroom conditions as perceived by school students, rather than relying exclusively on student teachers' self‐reports, the study reduces the risk of inflated correlations due to common method variance (Podsakoff et al., 2003). This approach also enhances the ecological validity of the findings, offering a more comprehensive view of the classroom environment and its impact on emotions in a sample of student teachers who may not yet possess a fully developed professional vision of classroom events (Weber et al., 2018).

However, the study is not without limitations. The analysis focused on within‐person relationships, but with only three measurements per person, the study falls short of the recommended number of observations for diary studies, which typically suggest five daily measures with 100 participants (Ohly et al., 2010). With such a low number of measurements, we may have not captured diverse teaching situations. The number of student teachers with no variation between the three measurement occasions for a specific study item ranged between 20% (for the item “I was satisfied with my own performance in this lesson” to capture control appraisals) and 48% (for the item “In this lesson it was very important to teach well” to capture value appraisals).

Another limitation is the heterogeneity of our sample, particularly in terms of prior teaching experiences and practicum formats. Over half of the student teachers reported teaching experience outside formal teacher education programmes, yet the specifics of these experiences remain largely unknown. Furthermore, practicum structures varied across institutions, ranging from intensive 3‐week units to part‐time commitments spread over a semester. While focusing on a 3‐week unit enhanced comparability, this variability may still limit the generalizability of our findings. Future research should consider more homogeneous sampling or conduct subgroup analyses to explore how different practicum conditions and prior experiences influence experiences during the teaching practicum.

Finally, the short scale used to assess value appraisals demonstrated low internal consistency, which may have affected the reliability of the findings related to this construct. The standardized coefficients for the relation between value appraisals and enjoyment (*β* = .33, *p* = .06) and anxiety (*β* = .43, *p* = .30) were in the expected direction, suggesting a potential trend that may not have reached statistical significance due to a high standard error and limited sample size. Future studies should employ situational approaches with more frequent measurement occasions and refine the assessment tools to enhance reliability and statistical power.

## CONCLUSION

The findings of this study underscore the complexity of emotional experiences during the teaching practicum and highlight the need for targeted support and interventions to help student teachers navigate these challenges. The study calls for future research to explore interventions that balance the demands of teaching with the need to support student teachers' emotional well‐being, ultimately contributing to more effective teacher education programmes.

## AUTHOR CONTRIBUTIONS

**Eva Susann Becker:** Writing – original draft; methodology; validation; visualization; writing – review and editing; software; formal analysis; project administration; data curation. **Tina Hascher:** Conceptualization; funding acquisition; writing – review and editing; resources; project administration; supervision. **Thomas Goetz:** Writing – review and editing. **Fritz C. Staub:** Conceptualization; funding acquisition; writing – review and editing; supervision; resources; project administration.

## CONFLICTS OF INTEREST

The authors declare no conflicts of interest.

## Data Availability

Data are available upon request from the corresponding author.
